# Temporomandibular Disorder Treated With Chiropractic Therapy

**DOI:** 10.7759/cureus.36377

**Published:** 2023-03-19

**Authors:** Eric Chun-Pu Chu, Wai Ting Lee, Cherie Chau, Emmanuel Wong, Hay Yeung Cheng

**Affiliations:** 1 Department of Chiropractic, New York Medical Group, EC Healthcare, Kowloon, HKG

**Keywords:** tmj imaging, temporomandibular joint-tmj, tmj dysfunction, and tmj related problems, tmj pain, temporomandibular joint (tmj) disorders, tmj disorders, chiropractic therapy, chiropractic management

## Abstract

Temporomandibular disorders (TMDs) are common and affect the temporomandibular joint (TMJ) and surrounding musculoskeletal tissues. Although traditional rehabilitative treatments such as physiotherapy, occlusal splints, orthodontics, and electrotherapy effectively manage TMDs, chiropractic therapy is emerging as a promising non-invasive treatment option. We report a 39-year-old female patient with TMD who underwent chiropractic therapy, including spinal adjustments, soft tissue therapy, and exercise rehabilitation. After four weeks of treatment, the patient reported a complete resolution of symptoms and an improved quality of life score. Thereafter, the patient continued chiropractic treatment monthly for six months, during which she reported no symptoms and demonstrated improvements in her spinal range of motion, open-mouth anatomy, and cervical lordosis. This case study highlights the efficacy of applying an interdisciplinary approach to treating TMD and the potential of chiropractic therapy as a valuable treatment option for managing TMD.

## Introduction

Temporomandibular disorders (TMDs) constitute pathological symptoms that affect the temporomandibular joint (TMJ) and surrounding musculoskeletal tissues [1]. Previously, TMD was known as TMJ dysfunction syndrome, functional TMJ changes, myofascial pain dysfunction syndrome, and temporomandibular pain dysfunction syndrome. Annually, TMD occurs in 4% of the adult population in the USA. TMD is primarily characterized by musculoskeletal pain syndromes which comprise myofascial pain, myalgia, and arthralgia. [2]. The etiology of TMD is not fully understood; however, it is believed to involve a combination of biological, environmental, social, emotional, and cognitive factors. Common comorbidities include chronic headaches, fibromyalgia, autoimmune disease, sleep apnea, and psychiatric illness [3]. Interestingly, depression and anxiety have been found to increase TMD risk (risk ratio [RR]: 2.1) and myofascial pain (RR: 1.8) in a large prospective cohort study. Smoking also increased TMD risk in females under the age of 30 (RR=1.8) [3]. The complex interplay between the aforementioned factors contributes to the wide range of TMD presentations and the diverse number of potential treatment responses possible [3]. Pain accompanied by functional impairments and noisy joints are the most common symptoms of TMD [1]. Non-invasive rehabilitative treatment options relieve pain in 40-90% of patients, including physiotherapy, occlusal splints, orthodontics, and electrotherapy [4, 5]. Non-steroidal anti-inflammatory drugs (NSAIDs), such as naproxen, are most commonly prescribed for acute pain in TMD. [6]. Muscle relaxants and tricyclic antidepressants may also be used to treat muscle spasms and chronic pain, respectively [7]. Botulinum toxin (BTX), a potent neurotoxin produced by Clostridium botulinum bacteria, has also been investigated for TMD treatment [8]. Lastly, surgery is the final option in cases where rehabilitative techniques are not effective. Chiropractic therapy is a promising non-invasive approach that has been shown to manage TMD symptoms effectively [9, 10]. In a recent meta-analysis of 20 randomized controlled trials, chiropractic therapy was shown to significantly improve measures of pain, maximum mouth-opening (MMO), and disability in TMD patients [9]. Furthermore, spinal manipulation and manual mandibular therapy were the most effective chiropractic techniques to treat TMD [10]. However, no study to date has investigated the changes in anatomy post-therapy. To the best of our knowledge, we are the first to compare cervical and MMO anatomical structures before and after chiropractic therapy. This case study provides valuable insights regarding the efficacy of structural improvements in chiropractic treatments for TMD and serves as a foundation for future research in this area.

## Case presentation

**Figure 1:**
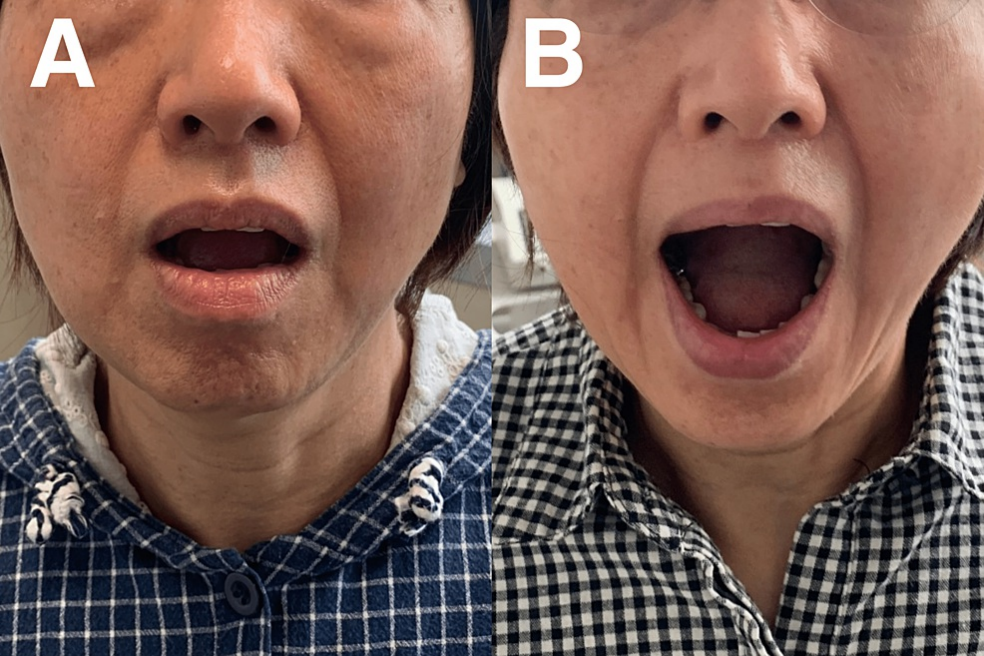
Maximum mouth-opening (MMO) measurements before and after therapy. A) Pre-treatment MMO at 30 mm; B) Post-treatment MMO at 50 mm.

A 39-year-old female presented with a six-month history of worsening bilateral pain and TMJ dysfunction. The pain was described as an ache in the preauricular area and temple, exacerbated by chewing, yawning, and talking. The patient reported frequent jaw locking in an open-mouth position, and the pain was rated at 6/10 in the numeric pain score. The patient further reported crepitus and clicks during jaw movement. MMO measurements decreased by 30 mm (Figure 1). Dental examination and panoramic radiography ruled out any dental pathology. The use of a nightguard did not relieve symptoms. The patient had no notable medical or dental history, including trauma, surgery, or other chronic health conditions.

**Figure 2:**
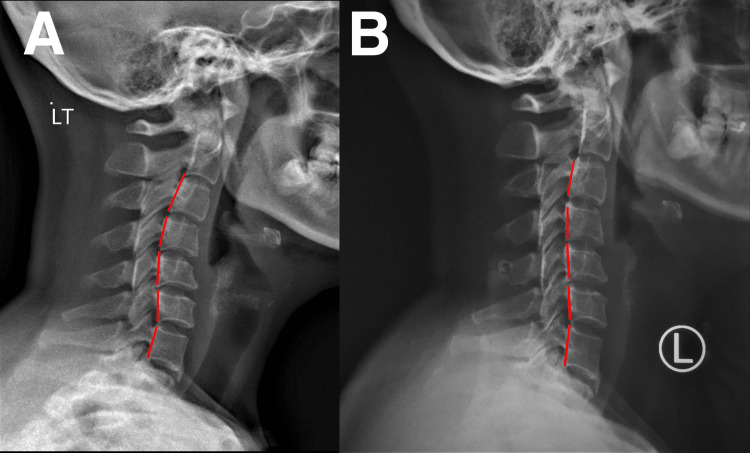
Cervical radiographs before and after therapy. A) Pre-treatment. Cervical deformity of reversed cervical lordosis at C1-4; B) Post-treatment. Cervical lordosis was reduced at C1-4.

**Figure 3:**
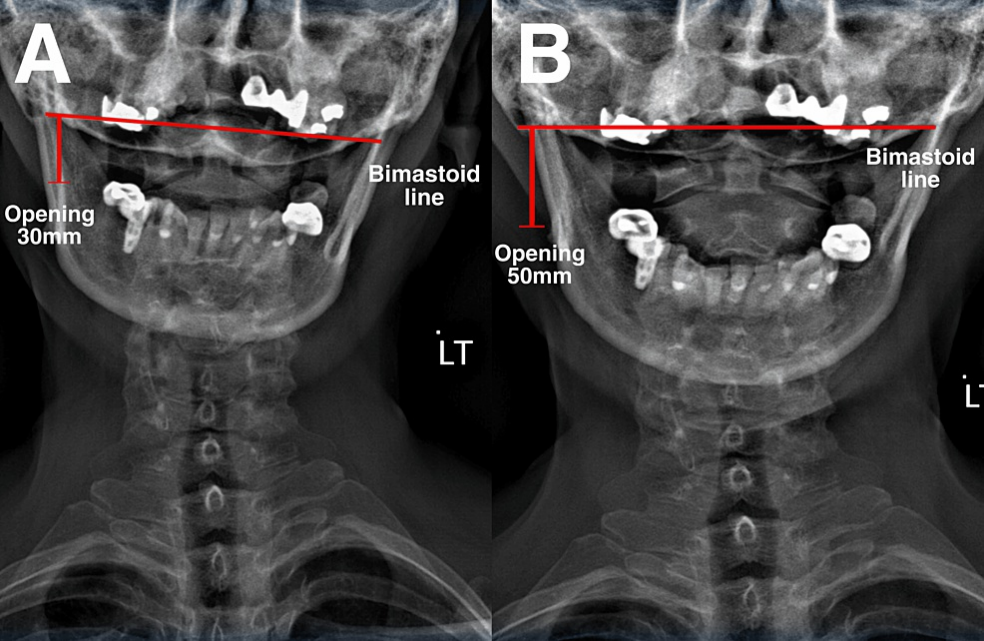
Anterior posterior open-mouth radiographs before and after therapy. A) Pre-treatment. Unbalanced bimastoid line and MMO of 30 mm; B) Post-treatment. Balanced bimastoid line and MMO of 50 mm. MMO: Maximum mouth opening.

Four weeks prior to the examination, the patient reported experiencing headaches, ear aches, and neck pain, which she believed was associated with jaw dysfunction. Chronic tension-type headaches appeared 3-4 times per week and ranked 6/10 on the numeric pain score. The pain affected the patient’s daily life, including eating, speaking, and sleeping. The patient was initially diagnosed with bilateral internal TMJ derangement with myofascial pain and dysfunction. The patient was referred to a chiropractor through her dentist. Upon examination, the patient exhibited tenderness and pain in the TMJ, with clicking and popping sounds during movement. No signs of swelling or inflammation were observed. The patient’s jaw had a limited range of motion and exhibited muscle tension in the neck and shoulders. Her cervical range of motion was considerably low at 15° compared to normal (>45°). Tenderness to palpation was reported in the bilateral TMJs, medial and lateral pterygoids, temporalis, masseter, sternoclavicular, and upper trapezius muscles. The maximum incisal opening or MMO was 30 mm, with lateral deviation upon opening. Unassisted opening and closing of the jaw were accompanied by crepitus and clicking. Cervical examinations revealed intersegmental dysfunction at C1/2, C5/6, and C7/T1; the neurological examination was unremarkable. Cervical (Figure 2) and anterior-posterior open-mouth (Figure 3) radiographs showed reduced lordosis and degenerative joint disease. Based on the physical examination and radiological findings, the patient was finally diagnosed with TMD and myofascial pain syndrome.

Chiropractic techniques used to treat TMD in our patient included spinal adjustment, soft tissue therapy, and exercise rehabilitation. Therapy was administered thrice a week for two weeks. Manual craniomandibular therapy was used to improve jaw mobility. Spinal manipulation at C1/2, C5/6, and C7/T1 to alleviate muscle tension and joint dysfunction. Scraping therapy was used to reduce soft tissue tightness in the neck and jaw. Lastly, exercise rehabilitation was used to improve the range of motion and strengthen the jaw and neck muscles. At the end of the two-week program, the patient reported a 70% improvement in TMD symptoms, including alleviation in jaw pain, clicking, and popping, as well as reduced headache and neck pain. The patient also reported an improved range of motion in the jaw and increased neck and shoulder mobility. Therapy administration was subsequently adjusted to twice a week. After four weeks, the patient fully recovered, and MMO was normal. The patient also regained a full score in their quality of life. Thereafter, the patient attended 1-2 sessions of chiropractic therapy per month for six months. On the sixth month of re-evaluation, she reported no symptoms and was able to open her mouth and chew more comfortably without pain or clicking sounds (Figure 1B). Her spinal range of motion also returned to normal. The radiographs showed improved open-mouth width (Figure 2B) and head/neck posture (Figure 3B). Overall, the patient showed positive results in numeric pain score, TMD, headaches, MMO, and cervical lordosis. 

## Discussion

Effective management of TMD requires a comprehensive understanding of the underlying pathoanatomic factors at play [10]. There is currently no gold standard for treating TMD [11]. Conservative treatment strategies that can best reduce the pain and disability associated with TMD depend on TMD etiology, severity, and patient preferences. Since TMD has a complex multifactorial etiology, we used a multimodal approach, including manual mandibular therapy, cervical spinal manipulation, and soft tissue therapies, demonstrating positive results. Manual mandibular therapy of the TMJ is regarded as the most effective treatment for alleviating pain and disability [10]. A recent systematic review reported that chiropractic therapy directed at the craniomandibular structures consistently improved measures of pain and MMO [12]. Although it would be great to match the best treatment option to the appropriate clinical presentation, such as acute or chronic, or optional therapy for patient characteristics such as age and sex, the gold standard of TMD therapy was unknown. Successful therapy selection appears most likely to influence anatomical structures directly associated with the etiology of TMD, such as the joint capsule, articular disc, and muscles of mastication, particularly the superior and inferior head of the lateral pterygoid. [10]. Cervical spinal manipulation, a high-velocity, low-amplitude thrust manipulation approach, is another highly effective treatment for reducing TMD symptoms [10]. In a randomized controlled trial investigating TMD treatment strategies, upper cervical spinal manipulation combined with dry needling was shown to be more effective against pain and MMO impairment compared to alternatives, including splint therapy, diclofenac, and TMJ mobilization at the three-month follow-up [13]. Another randomized controlled trial that investigated the effects of adding cervical spinal manipulation to standard care for TMD also found that cervical spinal manipulation had better outcomes in improving TMD-associated pain, mouth opening, disability, fear avoidance, and global perceived effect [14]. However, a previous study found that thoracic spinal manipulation does not affect TMD [15]. As the previous case report found relief of atypical symptoms with correction of cervical and thoracic curvature [16], the chiropractor decided to provide the treatment to both cervical and thoracic correction to reach the maximum outcome. Soft tissue treatments, including exercise rehabilitation and scraping therapy, have not been well studied [10]. It is used to treat TMD because of its significant muscular components. The muscles involved in mastication can develop trigger points, tightness, and imbalance which contribute to the overall pain and limited jaw function in TMD. Scraping therapy has been reported previously to reduce muscle tightness [17]. A quasi-experimental study published in 2023 demonstrated that chiropractic therapy reduced pain, improved range of motion and function, and decreased TMD severity compared to exercise therapy [18]. In this study, 24 patients who were randomized to receive either manual or exercise therapy had better outcomes, including pain (Numeric Pain Rating Scale), function (Patient-Specific Functional Scale), condition severity (Fonesca Amnestic Index), and mouth opening (millimeter mouth opening) in the manual therapy group [18]. Therefore, soft-tissue therapies may be used as a complement and at-home solution to other more established methods.

Interdisciplinary approaches combining multiple treatment modalities have been found to be effective in treating TMD. A scoping review that evaluated the effectiveness of an interdisciplinary approach for TMD included six studies, suggesting that an interdisciplinary strategy integrating multiple modalities may benefit patients with TMD by reducing pain, disability, and occlusal issues and improving patient-perceived changes [19]. Chiropractors are trained to assess and diagnose musculoskeletal conditions [20]; hence, they can perform cervical and thoracic spinal manipulations to correct spinal deformity [21, 22]. When the cervical or thoracic spine is malpositioned or fixated, the resulting change in somatosensory differentiation and kinesiopathology could exacerbate TMJ strain since proper functioning of the craniocervical-mandibular complex depends on the interlinking biomechanics of the upper cervical spine, occiput, and temporomandibular joints. Chiropractors can rectify these changes by facilitating motion segment synchronization, thereby reducing nociceptive inputs that could otherwise potentiate masticatory myofascial pain and compromise temporomandibular joint-related sensorimotor control. An interdisciplinary approach is therefore warranted in patients with TMD [23]. Chiropractic therapy may also provide additional benefits to patients. Many studies found that chiropractic techniques, including Chiropractic BioPhysics (CBP) and activators, are highly effective in reducing stress and improving the quality of life in patients with TMD [24-27]. Chiropractors play a crucial role in the early diagnosis and treatment of TMD and can further prevent the development of chronic TMD and degeneration. They are experienced in comprehensively assessing musculoskeletal symptoms [28] and can target their underlying causes, such as joint dysfunction, muscle tension, and poor posture. As chiropractic therapy is non-invasive and is associated with low risks [29], it can be the treatment of choice for patients who wish to avoid surgery or medication. Through a patient-centric and holistic approach to treatment, chiropractors can play a major role in restoring the quality of life in their patients [23]. Our patient showed positive results from the reduction of pain score in pain from 6 to 0, remission of symptoms including TMD and chronic tension-type headaches, improvement of structural changes including MMO (from 30 mm to 50 mm), and cervical lordosis measurements. However, there are limitations to the research due to its nature as a case study. Generalizing the results to a larger population without additional research is challenging. The report lacks a control group or comparison to other treatment options, making it difficult to determine whether the improvement was solely due to chiropractic therapy. The report lacks information provided about the patient's pre-existing medical conditions and any potential medication use, which could have contributed to the observed improvement. Therefore more rigorous research with larger sample sizes is needed to validate the conclusions.

## Conclusions

Our case study provides substantial evidence that chiropractic therapy is an effective treatment option for TMD and highlights the importance of imaging (photographs and cervical radiographs) to document changes in biomechanics to symptom alleviation. Further research is needed to fully assess the efficacy of different therapies for TMD. Nonetheless, our case study contributes enormously to the growing body of evidence supporting a multimodal treatment approach.
